# Longitudinal associations between ability in arts activities, behavioural difficulties and self-esteem: analyses from the 1970 British Cohort Study

**DOI:** 10.1038/s41598-019-49847-x

**Published:** 2019-10-02

**Authors:** Hei Wan Mak, Daisy Fancourt

**Affiliations:** Department of Behavioural Science and Health, University College London, London, UK

**Keywords:** Population screening, Epidemiology

## Abstract

Arts engagement has been shown to have benefits for young people’s psychological and behavioural adjustment. However, it is unknown whether it is frequency of arts engagement or individual ability in arts activities that is associated with these benefits. This study therefore examines the link between arts ability and children’s behavioural difficulties and self-esteem independent of frequency of engagement. We analysed data from the 1970 British Cohort Study with an overall sample size of 7700 for the behavioural difficulties outcome, and of 4991 for the self-esteem outcome. Baseline measures were taken when the children were aged 10 and followed up at age 16. OLS regression analysis adjusted for identified confounders shows that ability in the arts at age 10 was associated with a lower level of behavioural difficulties at age 16 independent of baseline behaviours, identified confounders and frequency of arts engagement. An association between arts ability and self-esteem was only found amongst children who have higher educational ability. These result suggest that there may be a value to encouraging the cultivation of arts skills at the onset of adolescence as a way of helping to foster children’s positive behavioural development.

## Introduction

A number of studies have identified a relationship between engagement in arts activities and young people’s psychological and behavioural adjustment, including subjective and psychological well-being, prosocial attitudes and behaviours, and self-esteem^1–5^. However, it remains unclear whether it is *frequency* of arts engagement or individual *ability* in arts activities that is associated with such benefits. A number of studies have shown that frequency of engagement in arts activities can lead to enhanced individual ability, referring to a person’s skill in performing artistic activities, such as painting, drawing, making models, and playing an instrument. For example, practice alone or with others is positively associated with acquiring practical skills for musicians^6^, and vocal training is associated with increased singing ability^7^. However, other studies have questioned this, suggesting that frequency of engagement and ability are in fact not causally linked, but could both be affected by other factors including quality of engagement (rather than mere hours spent)^8,9^, individual genetic variation^10^, and social factors such as parental support and the teacher’s characteristics^11^. It is therefore important to understand if there is a link between ability in the arts and children’s psychological and behavioural adjustment independent of frequency of engagement.

In considering *how* ability could be associated with young people’s behavioural and psychological adjustment, two theories provide a framework. The General Theory of Deviant Behaviour proposed by Kaplan^12,13^ suggests that deviant behaviours (e.g. bullying, stealing) are responses to negative self-attitudes. Negative self-attitudes arise if (a) people’s important others (e.g. family, peers) have negative attitudes towards them, (b) their ability to cope with self-devaluing experiences is poor, and (c) they do not achieve success in things they value. Individual ability in the arts may help to counter all three of these. First, ability may draw respect from family and peers as well as helping more broadly in the development of social connections and social cohesion^14,15^. Second, arts activities help enhance self-empowerment and self-worth in people’s own abilities^16^, which are particularly useful for developing social resilience and tackling challenges in other aspects of their lives^17^. Third, in common with almost all other abilities (e.g. reading and mathematical ability), a high level of arts ability could give rise to a strong sense of accomplishment. For instance, previous research has found a correlation between positive self-perceived musical skills, continuation of music participation and a feeling of achievement^18,19^. Further, it has been suggested by Bandura’s Self-Efficacy Theory^20^ that people with high levels of confidence in their ability (such as in the arts) have a greater sense of self-esteem. So it is plausible that the development of individual ability in the arts could translate into psychological and behavioural benefits.

In this study, therefore, we explored the longitudinal association between arts ability at age 10 and both behavioural difficulties and self-esteem at age 16: a sensitive developmental period as individuals who experience behavioural and psychological difficulties at this age may be at substantially elevated risk of deviant behaviours and mental disorders later in life^21–23^. Further, we explored whether this relationship was maintained even when considering frequency of arts engagement.

## Results

### Demographics

We used data from the 1970 British Cohort Study (BCS70), a prospective longitudinal survey. This study used Waves 3 (1980) and 4 (1986) interviews when the children were aged 10 and 16 respectively. A total of 10,651 participants and their parents provided data in both waves, of whom 7,700 provided data for the outcome measure of behavioural difficulties (measured using the Rutter Behaviour Scale) and 4,991 provided data for the outcome measure of self-esteem (measured using the Lawrence’s Self-Esteem Questionnaire). Multiple imputation was used to account for missing data on covariates to maintain this sample size. Of these participants, 49% were females and 96% of them were White. On average, 60% of the cohort had both parents who were working (see Table 1).

**Table 1:** Descriptive statistics.

Variables		Mean (SE)/%
**Outcome (age 16):** Child behaviours	Rutter behaviour scale (unstandardized; ranges from 20 to 60)	23.4 (4.12)
	Self-esteem (unstandardized; ranges from 0 to 20)	6.14 (8.38)
**Exposure (age 10):** Arts ability	Arts ability (unstandardized; ranges from 0 to 100)	58.2 (20.6)
**Covariates (age 10):** Gender	Female	49%
Ethnicity	White	96%
Parents’ employment status	No parents work	8.3%
	One parent works	32.3%
	Both parents work	59.5%
Household income	<£35–£49 per week	6.7%
	£50–£99 per week	30.1%
	£100–£149 per week	34.4%
	£150–£199 per week	16.5%
	£200–£250+ per week	12.4%
Parents’ socio-economic status	Professional	5.7%
	Intermediate	23.0%
	Skilled non-manual	10.7%
	Unskilled manual	41.7%
	Partly skilled	14.3%
	Unskilled	4.7%
Parents’ education	No qualification	51.9%
	O-level/A-level/Certificate of Education	26.2%
	Degree	10.2%
	Other qualification(s)	11.7%
Family composition	Intact family	21.1%
	Number of children in the household	2.47 (0.02)
Child’s educational ability	Higher education ability^a^	41.5%
	British Ability Scale: (Verbal) Word definitions	−0.17 (0.02)
	British Ability Scale: (Verbal) Word similarities	−0.12 (0.02)
	British Ability Scale: (Non- verbal) Recall of digits	−0.06 (0.02)
	British Ability Scale: (Non-verbal) Matrices	−0.23 (0.02)
	Reading ability	0.17 (0.01)
Physical activity	Freq. of physical activity^b^	2.42 (0.01)
Parent-child interactions	Mother’s interest in children’s education^c^	3.40 (0.01)
	Time spent on talking to the parents each day^d^	2.59 (0.00)
Mother’s mental health Child’s personalities and behaviour	Mother’s malaise score^e^	−0.12 (0.01)
	Child’s extroversion scale^f^	−0.02 (0.02)
	Child’s anxiousness scale^g^	−0.05 (0.02)
	Rutter behaviour scale^h^	420.01 (3.76)

Note: ^a^An additive score of various subjects, including maths, spelling, and creative writing. ^b^A three-point scale, “never/hardly ever”, “sometimes”, and “often”. ^c^A four-point scale, “uninterested”, “very little interested”, “moderate interested”, and “very interested”. ^d^A three-point scale, “none at all”, “not very much”, and “quite a lot”. ^e^Dervied from 22 items with a 1–100 scale (standardised). ^f^An introvert-extrovert scale rated from the teacher (standardised). ^g^An unworried-anxious scale rated from the teacher (standardised). ^h^The sum of 19 items with a visual analogue scale (each item ranges from 0 to 100).

### Arts ability and behavioural difficulties

We used OLS regression models to investigate the association between arts ability (measured as a standardised average of mothers’ ratings of their children’s ability in painting and drawing at home, making models, playing a musical instrument and reading music) and adolescents’ behavioural difficulties and self-esteem. Amongst the whole sample, a higher level of arts ability was associated with a lower level of behavioural difficulties in our unadjusted model (Table 2) (coef = −0.14, SE = 0.01, p < 0.001). This association was reduced by the inclusion of demographic factors (gender, ethnicity, parental employment status, household income, SES, parents’ education, family composition, and the number of children in the household), educational factors (children’s academic ability in maths, spelling, creative writing, reading, verbal ability and non-verbal ability as well as their physical activity), parent-child interactions (maternal interest in child’s education and the time spent on talking to the parents each day),and psychological factors (mothers’ malaise, child extroversion, child anxiety, and baseline behavioural difficulties). However, the relationship was still maintained even when controlling for all of these identified confounders (coef = −0.06, SE = 0.01, p < 0.001). This finding was consistent amongst children who had both lower (coef = −0.06, SE = 0.02, p = 0.001) and higher levels of arts engagement (coef = −0.05, SE = 0.02, p = 0.015).

**Table 2:** Relationship between arts ability (age 10) and behavioural difficulties and self-esteem (age 16): the whole sample and stratified sample by arts engagement frequency.

	Whole sample				Lower arts engagement frequency at age 10				Higher arts engagement frequency at age 10			
	Behavioural difficulties		Self-esteem		Behavioural difficulties		Self-esteem		Behavioural difficulties		Self-esteem	
	B ± SE	P	B ± SE	P	B ± SE	P	B ± SE	P	B ± SE	P	B ± SE	P
Model 1	−0.14 ± 0.01	<0.001	0.10 ± 0.02	<0.001	−0.14 ± 0.02	<0.001	0.07 ± 0.02	=0.001	−0.12 ± 0.02	<0.001	0.06 ± 0.03	=0.028
Model 2	−0.13 ± 0.01	<0.001	0.07 ± 0.02	<0.001	−0.13 ± 0.02	<0.001	0.05 ± 0.02	=0.025	−0.10 ± 0.02	<0.001	0.03 ± 0.03	=0.193
Model 3	−0.09 ± 0.01	<0.001	0.03 ± 0.02	=0.047	−0.09 ± 0.02	<0.001	0.03 ± 0.02	=0.182	−0.09 ± 0.02	< 0.001	0.01 ± 0.03	=0.816
Model 4	−0.06 ± 0.01	<0.001	0.02 ± 0.02	=0.235	−0.06 ± 0.02	=0.001	0.02 ± 0.02	=0.499	−0.05 ± 0.02	=0.015	−0.00 ± 0.03	=0.915
N	7700		4991		4622		2827		3020		2113	

Note: Statistical significance is denoted by asterisks: *sig at 5%, **sig at 1%, ***sig at 0.1%.

Model 1 was unadjusted, model 2 adjusted for demographic factors (gender, ethnicity, parental employment status, household income, SES, parents’ education, family composition, and number of children in the household), model 3 additionally adjusted for child academic ability (maths, spelling, creative writing, reading, verbal ability and non-verbal ability) as well as physical activity, while model 4 additionally controlled for parent-child interactions (mother’s interest in child’s education and the time spent on talking to the parents each day) and mental health (mothers’ malaise, child extroversion, child anxiety, and baseline behavioural difficulties).

Results were consistent when stratifying the sample by level of SES (lower SES: coef = −0.05, SE = 0.02, p = 0.010; higher SES: coef = −0.06, SE = 0.02, p < 0.001) and by level of educational ability (lower educational ability: coef = −0.05, SE = 0.02, p = 0.001; higher educational ability: coef = −0.05, SE = 0.02, p = 0.006) (Table 3). Further, findings were consistent in both boys and girls (boys: coef = −0.07, SE = 0.02, p < 0.001; girls: coef = −0.05, SE = 0.02, p = 0.003) (Supplementary Table 1). When excluding those with behavioural problems at baseline (the worst 20% of behavioural scores), results were also maintained (coef = −0.05, SE = 0.01, p < 0.001) (Supplementary Table 2). When using the categorical rather than linear scoring system for the Rutter scale which categorises children as having ‘normal’, ‘moderate’ or ‘severe’ behavioural problems, higher levels of arts ability at age 10 were associated with lower odds of developing severe behavioural difficulties by age 16 (coef = −0.22, SE = 0.08, p = 0.006) (Supplementary Table 3). Additionally, when exploring specific sub-components of behavioural problems, arts ability was associated with a lower probability of developing aggressiveness and hyperactivity, but the association with anxiety/fearfulness was attenuated when including baseline mental health (aggressiveness: coef = −0.03, SE = 0.01, p = 0.039; anxiety-fearfulness: coef = −0.02, SE = 0.01, p = 0.215; hyperactivity: coef = −0.06, SE = 0.01, p < 0.001) (Supplementary Table 4). Finally, when deconstructing the measure of arts ability into individual activities we found that the ability in making models (coef = −0.05, SE = 0.01, p < 0.001), playing a musical instrument (coef = −0.03, SE = 0.01, p = 0.016) and reading music (coef = −0.05, SE = 0.01, p < 0.001) were associated with a lower level of behavioural difficulties, but the association between painting and drawing at home and behavioural difficulties was attenuated after controlling for parent-child interactions and mental health factors (coef = 0.00, SE = 0.01, p = 0.984) (Supplementary Table 5).

**Table 3:** By parents’ SES and children’s education performance.

	Lower SES				Higher SES				Lower education				Higher education			
	Behavioural difficulties		Self-esteem		Behavioural difficulties		Self-esteem		Behavioural difficulties		Self-esteem		Behavioural difficulties		Self-esteem	
	B ± SE	P	B ± SE	P	B ± SE	P	B ± SE	P	B ± SE	P	B ± SE	P	B ± SE	P	B ± SE	P
Model 1	−0.11 ± 0.02	<0.001	0.04 ± 0.02	=0.046	−0.15 ± 0.02	<0.001	0.11 ± 0.02	<0.001	−0.14 ± 0.02	<0.001	0.06 ± 0.02	=0.002	−0.14 ± 0.02	<0.001	0.10 ± 0.02	<0.001
Model 2	−0.10 ± 0.02	<0.001	0.04 ± 0.02	=0.122	−0.15 ± 0.02	<0.001	0.10 ± 0.02	<0.001	−0.13 ± 0.02	<0.001	0.04 ± 0.02	=0.115	−0.12 ± 0.02	<0.001	0.09 ± 0.03	=0.001
Model 3	−0.08 ± 0.02	<0.001	0.01 ± 0.02	=0.758	−0.10 ± 0.02	<0.001	0.06 ± 0.02	=0.013	−0.09 ± 0.02	<0.001	0.01 ± 0.02	=0.645	−0.08 ± 0.02	<0.001	0.06 ± 0.03	=0.015
Model 4	−0.05 ± 0.02	=0.010	−0.01 ± 0.02	=0.819	−0.06 ± 0.02	<0.001	0.05 ± 0.02	=0.055	−0.05 ± 0.02	=0.001	−0.01 ± 0.02	=0.725	−0.05 ± 0.02	=0.006	0.06 ± 0.03	=0.035
N	3277		2254		4356		2679		4334		2784		3289		2136	

Note: Statistical significance is denoted by asterisks: *sig at 5%, **sig at 1%, ***sig at 0.1%.

Model 1 was unadjusted, model 2 adjusted for demographic factors (gender, ethnicity, parental employment status, household income, SES, parents’ education, family composition, and number of children in the household), model 3 additionally adjusted for child academic ability (maths, spelling, creative writing, reading, verbal ability and non-verbal ability) as well as physical activity, while model 4 additionally controlled for parent-child interactions (mother’s interest in child’s education and the time spent on talking to the parents each day) and mental health (mothers’ malaise, child extroversion, child anxiety, and baseline behavioural difficulties).

### Arts ability and self-esteem

There was a less consistent association between arts ability and self-esteem. Although arts ability and self-esteem were related in unadjusted models (coef = 0.10, SE = 0.02, p < 0.001), and even when controlling for demographic factors (coef = 0.07, SE = 0.02, p < 0.001) and educational factors (coef = 0.03, SE = 0.02, p = 0.047), when considering parent-child interactions, maternal and child mental health, and baseline behaviours, this association was attenuated (coef = 0.02, SE = 0.02, p = 0.235) (Table 2). Results in fully-adjusted models remained non-significant when splitting the sample by lower (coef = 0.02, SE = 0.02, p = 0.499) and higher arts engagement (coef = −0.00, SE = 0.03, p = 0.915) (Table 2).

When considering sub-group analyses, the relationship was found amongst those of higher educational ability (coef = 0.06, SE = 0.03, p = 0.035) and there were suggestions it was present in those of higher SES (coef = 0.05, SE = 0.02, p = 0.055), but it was not found amongst those of lower SES (coef = −0.01, SE = 0.02, p = 0.819) or lower educational ability (coef = −0.01, SE = 0.02, p = 0.725) (Table 3). When stratifying the sample by gender, no significant association was found between arts ability and self-esteem (boys: coef = 0.01, SE = 0.03, p = 0.827; girls: coef = 0.03, SE = 0.02, p = 0.206) (Supplementary Table 1). There was also no evidence for the association when excluding those with baseline behavioural problems (coef = −0.01, SE = 0.02, p = 0.958) (Supplementary Table 2). When deconstructing type of arts activity, there was no association between any of the individual arts activities and children’s self-esteem after controlling for all covariates (Supplementary Table 5).

## Discussions

Prior research has often shown that arts engagement is associated with better psychological and behavioural outcomes amongst young people, but most research has focused on frequency of engagement. This study went beyond this and investigated the relationship between arts *ability* and adolescents’ behavioural difficulties and self-esteem. We found that arts ability at age 10 is significantly, inversely associated with behavioural difficulties at age 16. It is of note that factors such as demographics, SES, child academic abilities, child mental health and maternal mental health explained a large proportion of this association. This is as expected, given that such factors are linked both with access to the arts and also with child mental health^24–26^. However, results were maintained independent of these factors. In particular, the association between arts ability and behavioural difficulties remains significant amongst children with lower levels of arts engagement frequency. This suggests that arts ability may have an independent relationship with positive behavioural development in childhood.

On the contrary, we found less evidence of a relationship between arts ability and self-esteem. This echoes findings from previous research that showed no association between music, art or reading ability and self-esteem at age 11^27^. However, it is of note that we did find a relationship in our sub-group analyses for children with stronger educational ability at age 10. Previous research has shown a relationship between positive self-worth and school grades, with a further relationship with lower delinquency^28^. So it is possible that, although arts ability may not be related to self-esteem amongst all children, amongst those who are already showing strong academic achievement (and related self-esteem), arts ability may have additive benefits. However, this remains to be explored further.

This study has several limitations. First, causality cannot be assumed from these observational findings. However, we did use a large and nationally-representative dataset, longitudinal tracking over 6 years, and we controlled for all identified confounding factors at baseline. Second, while we aimed to disentangle frequency of engagement from ability by stratifying the sample, it is reasonable to believe that some aspects of behavioural difficulties and self-esteem may still be influenced by frequency of engagement, rather than the arts ability alone. Future studies could build on these findings by using advanced techniques (e.g. propensity score matching) to disentangle the relationship between arts ability and frequency of arts engagement. We also believe there is scope for a better understanding of the Rutter Behaviour Scale’s underlying dimensions; our supplementary analysis suggest that arts ability may exert greater influence on children’s aggressiveness and hyperactivity than their anxiety-fearfulness, but these three latent variables extracted from a factor analysis only accounted for 40% of the variance. Whilst this was in line with previous factor analyses of the scale, further work exploring these separate constructs in more detail using other data could help to elucidate what the potential benefits of cultivating ability in arts activities are for child behaviours. We were also only able to examine children’s Rutter scores based on mothers’ reports, as teachers’ reports on the Rutter questions were not available in the data. It would be worthwhile to repeat the analyses using teacher’s reports, given these have been shown to produce more reliable estimates^29^.

Finally, our definition of arts ability was limited by what questions were available within BCS70. We were unable to explore children’s ability in other popular arts activities such as drama and dance. Our results suggest that average ability in arts activities and also specific ability in music and crafts are associated with positive behavioural development, but for painting and drawing results are attenuated when considering maternal mental health and child behaviours. Notably, there was an overall higher mean ability for painting and drawing and less variability in ability score, which could have led to a ceiling effect explaining the lack of sustained significance of the finding. But it is also possible that there are subtle distinctions between types of activity that influence behaviours. So future studies might wish to consider a wider range of arts activities when investigating the relationship between arts ability and children’s behavioural and psychological adjustment.

Overall, our results suggest there is an independent longitudinal relationship between arts ability and behavioural difficulties around the onset of adolescence, but not with self-esteem. These findings imply there could be a value to encouraging the cultivation of creative skills around the onset of adolescence as a way of helping to buffer against of aggression and hyperactivity and promoting positive behavioural development. Future intervention studies are therefore encouraged.

## Methods

### Participants

This study analysed data from the 1970 British Cohort Study (BCS70); a prospective longitudinal survey that follows a group of people (N = 17,000) from infancy into adulthood (age 50). Data were drawn from Waves 3 (1980) and 4 (1986) interviews when the children were aged 10 and 16 respectively. A total of 10,651 participants and their parents provided data in both waves. Of these, around 72% of the parents were given the questionnaire for behavioural difficulties at Wave 4 (N = 7700) and 3706 participants provided full data across all other measures that were included in analyses. With respect to the self-esteem items, only 50% of the cohort members were given the questionnaire for self-esteem (N = 4991) in the Wave 4 interview and, of these, 1922 provided full data across all other measures included in the analyses. The reduction in sample size across both measures was largely due to cohort members having left school, and industrial action by the teachers, resulting in a delay of the survey and incomplete data collection^30^. So multiple imputation was used to account for missing data, providing an overall sample size of 7700 for the Rutter Behaviour scale outcome and 4991 for the self-esteem outcome.

BCS70 has received ethical approval from the NHS Multi-Centre Research Ethics Committee (MREC) and all participants gave informed consent. All methods were performed in accordance with the relevant guidelines and regulations.

### Measures

We used the Rutter Behaviour Scale reported by mothers to measure children’s behaviour at home and at school: a well-validated instrument that was originally developed for use with British children/adolescents and has been used in epidemiological and psychological studies to detect behavioural problems in young people^31^. Our main model was based on an additive scale derived from 20 items, in which parents were asked to give descriptions of children’s behaviours (i.e. “does not apply”; “applies somewhat”; “definitely applies”). The scale was then standardised to have a mean of zero and a standard deviation of 1, with higher scores indicating a greater incidence of behavioural difficulties.

For self-esteem, we used the Lawrence’s Self-Esteem Questionnaire (LAWSEQ) to measure children’s self-reported sense of self-worth and self-esteem^32^. The scale was derived from 10 items, ranging from 0 (the lowest self-esteem score) to 20 (the highest self-esteem score), and standardised.

Children’s abilities in arts at age 10 were rated by their mothers on a scale from 0 to 100. We created an index of average ability derived from 4 ability scales - painting and drawing at home, making models, playing a musical instrument, and reading music. The scale was averaged and then standardised.

We identified a number of potentially confounding factors that we controlled for at baseline (age 10). For demographic and socio-economic factors, we included parent-reported gender, ethnicity (white or non-white), parental employment status (no parents work, one parent works, or both parents work), household income (£ per week), socio-economic status (SES; professional, intermediate, skilled non-manual, unskilled manual, partly skilled, or unskilled), parents’ education (no qualification, O-level/A-level/Certificate of Education, degree, or other qualification(s)), family composition (intact or non-intact family), number of children in the household, mothers’ malaise score, and children’s frequency of physical activity. For child academic ability, we included both subjective and objective measures including children’s self-reported academic abilities (binary scores assessing whether children felt they did well vs did not do well in maths, spelling, and creative writing; summed and then binarised into the top 40% of responses vs other responses), parent-reported child reading ability (a standardised scale of an average of three reading-related abilities rated by the mother: recalling the content of a book, making use of public library, and reading comics and magazines), and objective academic ability using the British Ability Scales (BAS) at age 10^33^. This scale has four academic ability subscales: word definitions and word similarities (to measure verbal ability), and recall of digits and matrices (to measure non-verbal ability).

We also adjusted for several variables that captured the interactions between children and their parents: mother’s interest in child’s education (reported by teachers) and the time that children reported spending talking to their parents each day. Additionally, we controlled for children’s mental health and behaviours at baseline: the degree of extroversion (rated by teachers; standardised), the level of anxiety (rated by teachers; standardised), and behavioural difficulties measured using the Rutter behaviour scale (rated by mothers; a visual analogue scale with a mean score of 420).

### Statistics

To account for missing values and attrition between waves, multiple imputation by chained equations using all variables included in our statistical models was carried out to create 50 imputed data sets. We used *regress*, *logit* and *mlogit* commands within the *mi* command to impute linear, binary and categorical variables respectively. Missingness across variables is shown in Supplementary Tables 6 and 7. Sensitivity analyses using the available data produced very similar results, so we present results from the imputed data sets for greater statistical power.

OLS regression models were used to investigate the association between arts ability and adolescents’ behavioural difficulties and self-esteem. In order to identity possible underlying mechanisms that may explain the association, we built our models sequentially. Model 1 was unadjusted, model 2 adjusted for demographic factors (gender, ethnicity, parental employment status, household income, SES, parents’ education, family composition, number of children in the household), model 3 additionally adjusted for child academic ability (maths, spelling, creative writing, reading, verbal ability and non-verbal ability) as well as physical activity, while model 4 additionally controlled for parent-child interactions (mother’s interest in child’s education and the time spent on talking to the parents each day) and mental health (mothers’ malaise, child extroversion, child anxiety, and baseline behavioural difficulties). The risk of multicollinearity was very low with a mean value of the Variance Inflation Factor (VIF) of 1.22, suggesting that our analyses did not violate the OLS assumptions. All other model assumptions were also met. Given we had two outcome variables, a Bonferroni correction with an alpha of 0.05/2 (outcomes) = 0.025 can be applied to assess the significance of results after adjustment for multiple comparisons.

Given that arts ability and frequency of arts engagement was highly correlated (r = 0.51), in addition to analysing the whole sample, we also stratified the sample into two sub-groups using a median split: lower arts engagement frequency and higher arts engagement frequency. Frequency of arts engagement was measured by reports from mothers as to the frequency with which their child engaged in various arts activities, including going to a museum and playing a musical instrument.

As supplementary analyses, we repeated the analyses stratified by (1) parents’ SES, (2) children’s educational performance, and (3) children’s gender to assess whether there were any sub-group differences. Additionally, to assess whether results were skewed by inclusion of those with behavioural difficulties at baseline, we carried out the analyses by excluding the respondents who were at the top 20% on the Rutter Behaviour Scale (i.e. those with the highest behavioural difficulties). We also tested whether using the alternative scoring system for the Rutter scale affected results by additionally presenting models using the categorical indicator of behaviours which divides children into three subgroups- (a) “normal”, scores below the 80^th^ percentile; (b) “moderate”, scores between the 80^th^ and 95^th^ percentile; and (c) “severe”, scores above the 95^th^ percentile^34^). In order to identify any potential underlying sub-categories of the Rutter Behaviour Scale, we also performed a factor analysis and extracted three factors (as also were found in McGee *et al*.‘s study^35^): aggressiveness (Cronbach’s α = 0.80), anxiety-fearfulness (Cronbach’s α = 0.68), and hyperactivity (Cronbach’s α = 0.62). The three-factor structure of Rutter Behaviour Scale was confirmed by eigenvalues >1 and visual inspection of a screeplot according to Kaiser’s elbow criterion^36^ accounted for 40% of the variance (Kaiser-Meyer-Olkin = 0.88), in line with previous factor analyses of this scale^35^. Finally to test whether results were driven by average ability in any arts activity or a specific ability in a particular arts activity, we split our ability index into an individual ability score for each activity and re-ran analyses.

## Supplementary information

Supplementary tables

## Data Availability

The 1970 British Cohort Study data set is available via the UK Data Service.
