# Supplementary material for: Longitudinal associations between ability in arts activities, behavioural difficulties and self-esteem: analyses from the 1970 British Cohort Study
Source: Sci Rep. 2019 Oct 2;9:14236. doi: 10.1038/s41598-019-49847-x (PMC6775110; doi:10.1038/s41598-019-49847-x)
Supplement: Supplementary file 1 — Supplementary tables [file 41598_2019_49847_MOESM1_ESM.pdf]

Longitudinal associations between ability in arts activities, behaviour difficulties and self-esteem: analyses from the 1970 British Cohort Study

Hei Wan Mak[a] & Daisy Fancourt[a]*

[a]Department of Behavioural Science and Health University College London, UK

*Corresponding author: Daisy Fancourt, 1-19 Torrington Place, University College London, London WC1E 7HB

Supplementary table 1: by gender

	Boys				Girls			
	**Behavioural difficulties**		**Self-esteem**		**Behavioural difficulties**		**Self-esteem**	
	**B±SE**	**P**	**B±SE**	**P**	**B±SE**	**P**	**B±SE**	**P**
Model 1	-0.15±0.02	<0.001	0.09±0.03	<0.001	-0.17±0.02	<0.001	0.13±0.02	<0.001
Model 2	-0.12±0.02	<0.001	0.04±0.03	=0.178	-0.13±0.02	<0.001	0.09±0.02	<0.001
Model 3	-0.09±0.02	<0.001	0.02±0.03	=0.557	-0.10±0.02	<0.001	0.05±0.02	=0.042
Model 4	-0.07±0.02	<0.001	0.01±0.03	=0.827	-0.05±0.02	=0.003	0.03±0.02	=0.206
N	3764		2188		3936		2803	

Note: Statistical significance is denoted by asterisks: † sig at 10%, * sig at 5%, ** sig at 1%, *** sig at 0.1%.

Model 1 was unadjusted, model 2 adjusted for demographic factors (ethnicity, parental employment status, household income, SES, parents' education, family composition, and number of children in the household), model 3 additionally adjusted for child academic ability (maths, spelling, creative writing, reading, verbal ability and non-verbal ability) as well as physical activity, while model 4 additionally controlled for parent-child interactions (mother's interest in child's education and the time spent on talking to the parents each day) and mental health (mothers' malaise, child extroversion, child anxiety, and baseline behavioural difficulties).

Supplementary table 2: Exclude the top 20% behavioural problems

	Behavioural difficulties		Self-esteem	
	**B±SE**	**P**	**B±SE**	**P**
Model 1	-0.09±0.01	<0.001	0.06±0.02	<0.001
Model 2	-0.08±0.01	<0.001	0.04±0.02	=0.049
Model 3	-0.06±0.01	<0.001	0.01±0.02	=0.752
Model 4	-0.05±0.01	<0.001	-0.01±0.02	=0.958
N	5465		3512	

Note: Statistical significance is denoted by asterisks: * sig at 5%, ** sig at 1%, *** sig at 0.1%.

Model 1 was unadjusted, model 2 adjusted for demographic factors (gender, ethnicity, parental employment status, household income, SES, parents' education, family composition, and number of children in the household), model 3 additionally adjusted for child academic ability (maths, spelling, creative writing, reading, verbal ability and non-verbal ability) as well as physical activity, while model 4 additionally controlled for parent-child interactions (mother's interest in child's education and the time spent on talking to the parents each day) and mental health (mothers' malaise, child extroversion, child anxiety, and baseline behavioural difficulties).

Supplementary Table 3: Behavioural difficulties as threefold category using multinomial logistic regression (presented in log odds)

	**Moderate vs normal**		**Severe vs normal**	
	**B±SE**	**P**	**B±SE**	**P**
Model 1	-0.25±0.04	<0.001	-0.48±0.07	<0.001
Model 2	-0.18±0.05	<0.001	-0.47±0.07	<0.001
Model 3	-0.13±0.05	=0.009	-0.33±0.07	<0.001
Model 4	-0.08±0.05	=0.119	-0.22±0.08	=0.006
N	7700		7700	

Note: Statistical significance is denoted by asterisks: * sig at 5%, ** sig at 1%, *** sig at 0.1%.

Model 1 was unadjusted, model 2 adjusted for demographic factors (gender, ethnicity, parental employment status, household income, SES, parents' education, family composition, and number of children in the household), model 3 additionally adjusted for child academic ability (maths, spelling, creative writing, reading, verbal ability and non-verbal ability) as well as physical activity, while model 4 additionally controlled for parent-child interactions (mother's interest in child's education and the time spent on talking to the parents each day) and mental health (mothers' malaise, child extroversion, child anxiety, and baseline behavioural difficulties).

Supplementary Table 4: Behavioural difficulties factor structure

	Aggressiveness		Anxiety-fearfulness		Hyperactivity	
	**B±SE**	**P**	**B±SE**	**P**	**B±SE**	**P**
Model 1	-0.11±0.01	<0.001	-0.02±0.01	=0.193	-0.13±0.01	<0.001
Model 2	-0.08±0.01	<0.001	-0.06±0.01	<0.001	-0.09±0.01	<0.001
Model 3	-0.05±0.01	=0.001	-0.04±0.01	=0.007	-0.08±0.01	<0.001
Model 4	-0.03±0.01	=0.039	-0.02±0.01	=0.215	-0.06±0.01	<0.001
N	7700		7700		7700	

Note: Statistical significance is denoted by asterisks: * sig at 5%, ** sig at 1%, *** sig at 0.1%.

Model 1 was unadjusted, model 2 adjusted for demographic factors (gender, ethnicity, parental employment status, household income, SES, parents' education, family composition, and number of children in the household), model 3 additionally adjusted for child academic ability (maths, spelling, creative writing, reading, verbal ability and non-verbal ability) as well as physical activity, while model 4 additionally controlled for parent-child interactions (mother's interest in child's education and the time spent on talking to the parents each day) and mental health (mothers' malaise, child extroversion, child anxiety, and baseline behavioural difficulties).

Supplementary Table 5: Various kinds of arts ability

	Painting and drawing at home Mean 81.6 SD 18.1				Making models Mean 72.6 SD 27.1				Playing a musical instrument Mean 44.8 SD 36.8				Reading music Mean 34.0 SD 34.5			
	Behavioural difficulties		Self-esteem		Behavioural difficulties		Self-esteem		Behavioural difficulties		Self-esteem		Behavioural difficulties		Self-esteem	
	B±SE	P	B±SE	P	B±SE	P	B±SE	P	B±SE	P	B±SE	P	B±SE	P	B±SE	P
Model 1	-0.08± 0.01	<0.001	0.03± 0.02	=0.088	-0.14± 0.01	<0.001	0.08± 0.02	<0.001	-0.08± 0.01	<0.001	0.06± 0.02	<0.001	-0.11± 0.01	<0.001	0.08± 0.01	<0.001
Model 2	-0.07± 0.01	<0.001	0.02± 0.02	=0.261	-0.12± 0.01	<0.001	0.05± 0.02	=0.001	-0.07± 0.01	<0.001	0.05± 0.02	=0.006	-0.06± 0.01	<0.001	0.06± 0.02	<0.001
Model 3	-0.04± 0.01	=0.001	0.00± 0.02	=0.979	-0.09± 0.01	<0.001	0.03± 0.02	=0.066	-0.04± 0.01	=0.003	0.02± 0.02	=0.299	-0.05± 0.01	<0.001	0.03± 0.02	=0.055
Model 4	0.00± 0.01	=0.984	-0.01± 0.02	=0.637	-0.05± 0.01	<0.001	0.02± 0.02	=0.247	-0.03± 0.01	=0.016	0.01± 0.02	=0.543	-0.05± 0.01	<0.001	0.02± 0.02	=0.189
N	6995		4483		6995		4483		6995		4483		6995		4483	

Note: Statistical significance is denoted by asterisks: * sig at 5%, ** sig at 1%, *** sig at 0.1%.

SD: standard deviation. SE: standard error. Model 1 was unadjusted, model 2 adjusted for demographic factors (gender, ethnicity, parental employment status, household income, SES, parents' education, family composition, and number of children in the household), model 3 additionally adjusted for child academic ability (maths, spelling, creative writing, reading, verbal ability and non-verbal ability) as well as physical activity, while model 4 additionally controlled for parent-child interactions (mother's interest in child's education and the time spent on talking to the parents each day) and mental health (mothers' malaise, child extroversion, child anxiety, and baseline behavioural difficulties).

Supplementary table 6: Missing data for behavioural difficulties outcome (N=7700)

**Variables**	N	**% missing**
Arts ability	6728	12.6
Female	7700	0.00
White	7403	3.86
Parents' employment status	6386	17.1
Household income	6417	16.7
Parents' socio-economic status	6754	12.3
Parents' education	6521	15.3
Intact family	7536	2.13
Number of children in the household	6911	10.2
Higher education ability	6089	20.9
(Verbal) Word definitions	5864	23.8
(Verbal) Word similarities	5842	24.1
(Non- verbal) Recall of digits	5850	24.0
(Non-verbal) Matrices	5851	24.0
Reading ability	6995	9.16
Freq. of physical activity	6911	10.2
Mother's interest in children's education	5500	28.6
Time spent on talking to the parents each day	6273	18.5
Mother's malaise score	7601	1.29
Child's extroversion scale	6310	18.1
Child's anxiousness scale	6300	18.2
(Baseline) Rutter behaviour scale	6607	14.2

Supplementary table 7: Missing data for self-esteem outcome (N=4991)

**Variables**	N	**% missing**
Arts ability	4304	13.8
Female	4991	0.00
White	4761	4.61
Parents’ employment status	4087	18.1
Household income	4086	18.1
Parents’ socio-economic status	4332	13.2
Parents’ education	4191	16.0
Intact family	3962	20.6
Number of children in the household	4412	11.6
Higher education ability	3719	25.5
(Verbal) Word definitions	3704	25.8
(Verbal) Word similarities	3712	25.6
(Non- verbal) Recall of digits	3705	25.8
(Non-verbal) Matrices	4483	10.2
Reading ability	3898	21.9
Freq. of physical activity	4418	11.5
Mother’s interest in children’s education	3572	28.4
Time spent on talking to the parents each day	4018	19.5
Mother’s malaise score	3829	23.3
Child’s extroversion scale	4050	18.9
Child’s anxiousness scale	4034	19.2
(Baseline) Rutter behaviour scale	4227	15.3
